# THE CANNABIS AND OLDER PERSONS STUDY: WHAT WE HAVE LEARNED

**DOI:** 10.1093/geroni/igad104.0567

**Published:** 2023-12-21

**Authors:** Brian Kaskie

**Affiliations:** College of Public Health, University of Iowa, Iowa City, Iowa, United States

## Abstract

The Cannabis and Older Persons Study has examined the increasing use of cannabis among Americans over 60 years old since 2016 reflecting a variety of disciplinary perspectives and methodological approaches. The project team has published a dozen peer reviewed manuscripts and has secured competitive funding swards in California, Colorado, Illinois and Iowa. In this symposium, results from multiple COPS surveys collected from older adults are reviewed with three goals in mind. The first is to demonstrate how older cannabis users are not comparable to younger cannabis users in terms of motives and self-reported outcomes of use. The second is to distinguish therapeutic from recreational cannabis use among older persons by examining survey answers provided by persons with arthritis, cancer, caregivers of persons with Alzheimer’s disease, multiple sclerosis and terminal illnesses. The third goal is to differentiate life-time from naïve cannabis users and consider how cannabis may serve as an opioid avoidance or harm reduction strategy. Discussion considers the critical role policy makers, program administrators and clinicians assume in facilitating or hindering cannabis use, and directions for future research.

